# Enhancing 7-dehydrocholesterol suppresses brain ferroptosis and tissue injury after neonatal hypoxia–ischemia

**DOI:** 10.1038/s41598-024-58579-6

**Published:** 2024-04-04

**Authors:** Thiago C. Genaro-Mattos, Zeljka Korade, Namood-e Sahar, Jose Pedro Friedmann Angeli, Károly Mirnics, Eric S. Peeples

**Affiliations:** 1 Munroe-Meyer Institute for Genetics and Rehabilitation, University of Nebraska Medical Center, Omaha, NE 68106 USA; 2 Department of Pediatrics, University of Nebraska Medical Center, Omaha, NE 68198 USA; 3 Child Health Research Institute, Omaha, NE 68198 USA; 4 Rudolf Virchow Zentrum - Center for Integrative and Translational Bioimaging, University of Würzburg, Würzburg, Germany; 5 Department of Pediatrics, Children’s Nebraska, Omaha, NE 68114 USA

**Keywords:** Cellular neuroscience, Neonatal brain damage, Hypoxic-ischaemic encephalopathy

## Abstract

Neonatal hypoxic-ischemic brain injury (HIBI) results in part from excess reactive oxygen species and iron-dependent lipid peroxidation (i.e. ferroptosis). The vitamin D precursor 7-dehydrocholesterol (7-DHC) may inhibit iron-dependent lipid peroxidation. Primary neurons underwent oxygen and glucose deprivation (OGD) injury and treatment with 7-DHC-elevating medications such as cariprazine (CAR) or vehicle. Postnatal day 9 mice underwent sham surgery or carotid artery ligation and hypoxia and received intraperitoneal CAR. In neurons, CAR administration resulted in significantly increased cell survival compared to vehicle controls, whether administered 48 h prior to or 30 min after OGD, and was associated with increased 7-DHC. In the mouse model, malondialdehyde and infarct area significantly increased after HIBI in the vehicle group, which were attenuated by post-treatment with CAR and were negatively correlated with tissue 7-DHC concentrations. Elevating 7-DHC concentrations with CAR was associated with improved cellular and tissue viability after hypoxic-ischemic injury, suggesting a novel therapeutic avenue.

## Introduction

Neonatal hypoxic-ischemic encephalopathy, which is the clinical phenotype resulting from hypoxic-ischemic brain injury (HIBI), is a significant neurological injury occurring in the perinatal period. Every year, more than 1.1 million newborns are diagnosed with neonatal encephalopathy worldwide^1^. Currently, the only approved therapy is hypothermia; however, hypothermia has not shown to be beneficial in lower resource settings^2^ and despite treatment of patients in high-income countries, nearly half of the infants with moderate to severe HIBI die or suffer from significant developmental disability^3^. Given the high morbidity and mortality after neonatal HIBI despite available therapy, it is imperative that investigators continue to seek out novel supplemental therapies.

Neonatal HIBI is a multi-phasic injury. After reperfusion and restoration of appropriate oxygenation of the brain tissue, there is rapid production of free radicals and an increase in the oxidation of biomolecules, both of which are primary components in the secondary phase of HIBI^4^. Unsaturated lipids, polyunsaturated fatty acids, and sterols are prone to undergoing free radical-mediated lipid peroxidation. Lipid peroxidation is a key driver of a type of regulated cell death referred to as ferroptosis^5^. Ferroptosis has been linked to the pathophysiology of multiple disorders of ischemia and reperfusion, including intestinal ischemia^6^, myocardial infarction^7^, and ischemic stroke^8^. Additionally, ferroptosis was recently identified as a key mediator of cortical mitochondrial damage^9^ and hippocampal neuronal death^10^ after neonatal HIBI.

The lipid peroxidation occurring during ferroptosis opens pores in the cell membrane leading to calcium influx and ultimately cell death. This process can be inhibited by the addition of highly oxidizable chemicals, such as 7-dehydrocholesterol (7-DHC), that may prevent membrane lipids peroxidation^11^. Although chronic 7-DHC elevation is toxic^12–17^, it is the most oxidizable lipid known to date, being ~ 200 times more reactive than cholesterol and 6–10 times more reactive than the commonly studied antioxidants docosahexanoic acid (DHA) and arachidonic acid (ARA)^18,19^. Due to its oxidizability, 7-DHC likely also acts in a “sacrificial” role, shielding other vital phospholipids and thus preventing cell death. It is believed that the extra double bond in the sterol B ring of 7-DHC results in a more planar shape than cholesterol, allowing for increased interactions with phospholipid fatty acid side chains^20^. Supporting this theory, two recent preprints demonstrated that elevated 7-DHC leads to increased resistance to ferroptosis in cancer^21^ and hepatic ischemia–reperfusion^22^. The effects of 7-DHC elevation after neonatal HIBI remain unexplored, however.

**Figure 1:**
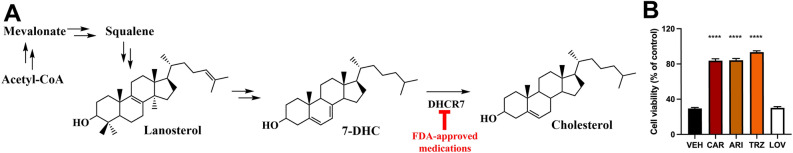
7-dehydrocholesterol (7-DHC)-elevating medications suppress RSL-3-mediated ferroptosis in HT1080. (**A**) denotes the simplified cholesterol biosynthesis pathway highlighting the inhibited reaction. (**B**) shows RSL-3 mediated ferroptosis in HT1080 cells pre-treated for 24 h with vehicle (VEH), 50 nM cariprazine (CAR), 500 nM aripiprazole (ARI), 500 nM trazodone (TRZ) or 50 nM lovastatin (LOV). Cell viability was assessed with alamarBlue. CAR, ARI and TRZ are potent DHCR7-inhibiting medications and significantly suppress ferroptotic induced cell death. LOV is a potent HMGCR inhibitor and it does not prevent ferroptosis, indicating that the ferroptosis suppression is not due to cholesterol deprivation, but to an increase in 7-DHC. Bars represent mean ± SEM of 6 replicates. ****p < 0.0001 in pairwise comparison between treatment and control (VEH).

Recent high-throughput screening studies of approximately 5000 chemicals revealed that 5% of FDA-approved medications increase 7-DHC in vitro, likely due to inhibition of 7-dehydrocholesterol reductase (DHCR7, Fig. 1A)^23,24^. Among the most active compounds were the psychotropic medications cariprazine (CAR), aripiprazole (ARI), and trazodone (TRZ). As such, this study sought to evaluate the effects of administering 7-DHC-elevating medication after brain hypoxia–ischemia in vitro and in a mouse model of neonatal HIBI. Our hypothesis was that elevating the concentration of 7-DHC in the brain during or after neonatal HIBI would result in decreased brain injury. These findings will allow for the development of future investigations seeking to target ferroptotic cell death in newborns with HIBI.

## Results

Using HT1080 cells, we assessed the in vitro effects of pre-treating cells with the 7-DHC-elevating medications CAR, ARI, and TRZ 24 h prior to RSL-3-induced ferroptosis. Administration of each medication resulted in significantly increased cell survival (83.7 ± 5.1% CAR; 84.1 ± 4.9% ARI; and 93.3 ± 3.7% TRZ) compared to vehicle controls (29.4 ± 2.7%, p < 0.0001 for each individual pairwise comparison, Fig. 1B). Additionally, we pre-treated HT1080 cells with lovastatin (LOV) – an inhibitor of 3-Hydroxy-3-Methylglutaryl-CoA Reductase (HMGCR) that results in decreased cholesterol but not elevation of 7-DHC – and demonstrated no significant change in cell survival (30.1% survival LOV) compared to vehicle controls (p = 0.998).

**Figure 2:**
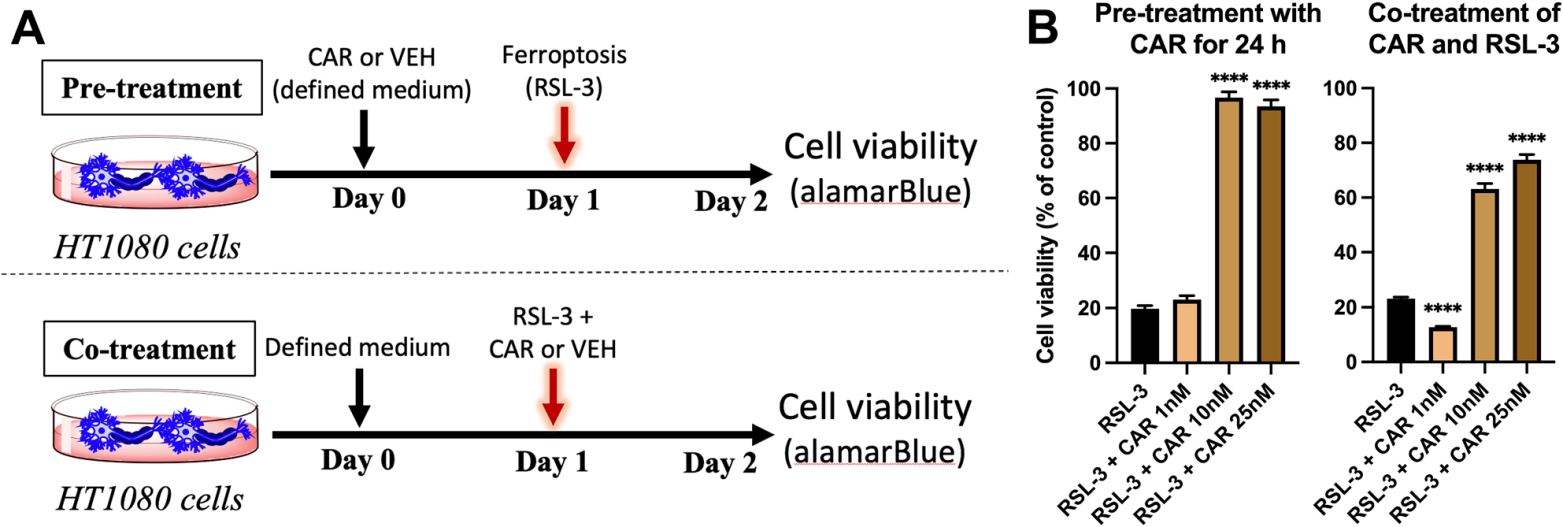
Cariprazine (CAR) attenuates RSL-3 induced ferroptotic cell death in a dose-dependent manner in HT1080 cells. (**A**) depicts the experimental design. (**B**) shows HT1080 cells pre-treated with 1, 10 and 25 nM CAR for 24 h before ferroptosis was induced with 25 nM RSL-3. Cells were incubated with RSL-3 for 24 h and cell viability was assessed with alamarBlue. Cell viability is significantly increased with CAR pre-treatment. Also shows HT1080 cells treated simultaneously with 25 nM RSL-3 and 1, 10 or 25 nM CAR for 24 h (co-treatment with CAR and RSL-3). Bars represent mean ± SEM of 16–24 replicates. Note that cell viability is significantly increased with CAR treatment. *p < 0.05 and ****p < 0.0001 in pairwise comparison between treatment and control (VEH).

Although the current study showed similar viability between the different drugs tested (Fig. 1B), previous studies demonstrated that CAR at the lowest concentrations resulted in the highest amount of 7-DHC elevation^25^, so it was chosen for the remaining experiments in this study to provide proof-of-concept. Treatment with different doses of CAR demonstrated a dose-dependent attenuation of RSL-3 induced ferroptotic cell death in HT1080 cells when administered 24 h prior to (Fig. 2B) or concurrent with (Fig. 2B) RSL-3 administration.

**Figure 3:**
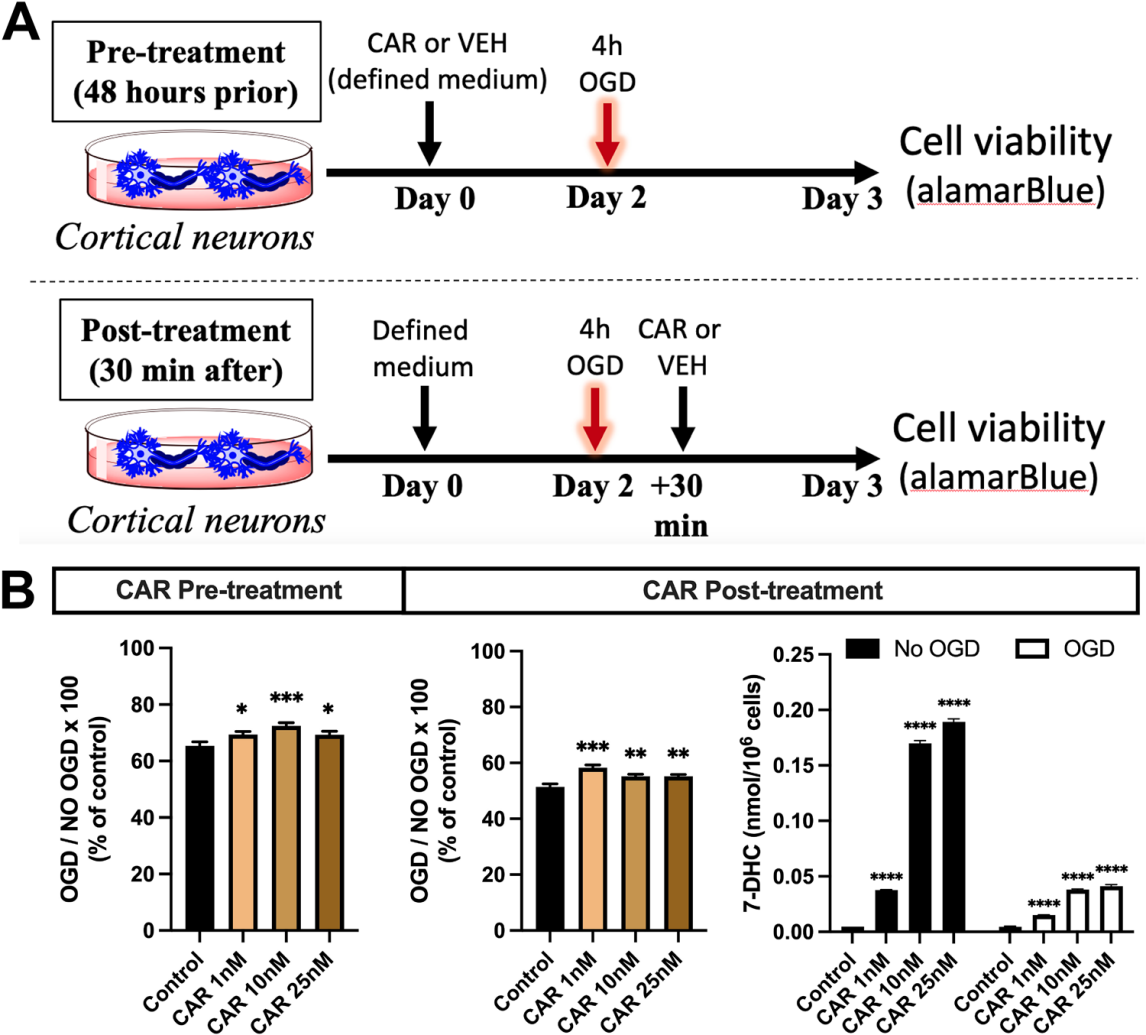
Cariprazine (CAR) preserves higher cell viability after oxygen–glucose deprivation (OGD) induced cell death in primary neurons. (**A**) shows the experimental design. (**B**) shows the effects of CAR on neuronal viability after 4 h of OGD. For the pre-treatment experiments, neurons were treated with vehicle, 1 nM, 10 nM or 25 nM CAR for 48 h before OGD. For the post-treatment experiments, neurons were treated with vehicle, 1 nM, 10 nM or 25 nM 30 min after OGD. Cell viability was assessed 24 h after the termination of OGD and was calculated by dividing the numbers of live neurons after OGD by the number of live neurons of the control (not exposed to OGD). 7-DHC levels were assessed by LC–MS/MS at the endpoint of the experiment. Concentrations of 7-DHC are elevated by CAR administration but consumed by OGD. *p < 0.05, **p < 0.01, ***p < 0.0001 in pairwise comparison between treatment and control (VEH). Bars represent mean ± SEM of 15–24 wells/group.

Next, we evaluated whether there was benefit to cell survival in the oxygen–glucose deprivation (OGD) model with primary neurons. There was increased cell survival at all doses of CAR whether administered 48 h prior to or 30 min after OGD (Fig. 3B); however, the magnitude of improvement was less in this more complex – but more translatable – cell death model and the dose-dependency was lost compared to the RSL-3 model. CAR administration did increase 7-DHC concentrations in the primary neurons in a dose-dependent manner in both the control and OGD cells (Fig. 3B). The 7-DHC was less at each dose of CAR in the OGD cells (p < 0.0001 between OGD and no OGD for each dose), which is consistent with oxidative consumption under the OGD conditions.

**Figure 4:**
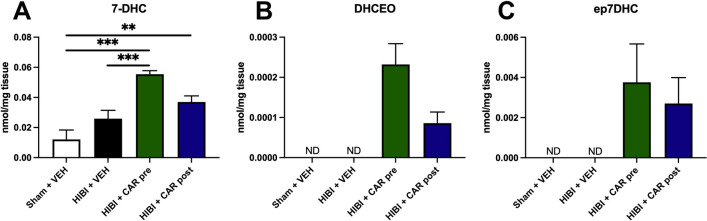
Cariprazine (CAR) pre- or post-treatment both resulted in an increase in 7-DHC and 7-DHC-derived oxysterols in the brain. 7-DHC derived oxysterols are increased in the brain tissue of CAR-treated animals after hypoxic-ischemic brain injury (HIBI). Animals were injected with 0.2 mg/kg of CAR as described in Fig. 5A. 7-DHC-derived oxysterols DHCEO and ep7DHC were not detected (ND) in the Sham + vehicle or HIBI + vehicle groups but were detected in both CAR groups. **p < 0.01, ***p < 0.001. Bars represent mean ± SEM of 7–10 replicates.

In the animal model of HIBI, CAR pre-treatment, but not post-treatment, resulted in significantly increased 7-DHC in the brain tissue compared to the HIBI vehicle group (Fig. 4A). Both pre- (0.06 ± 0.02 nmol/mg tissue, p < 0.0001) and post-treatment (0.04 ± 0.01 nmol/mg tissue, p = 0.006), however, had significantly higher 7-DHC concentrations compared to the sham controls (0.01 ± 0.02 nmol/mg tissue). These increases in 7-DHC were seen after CAR administration despite an increase in 7-DHC oxidation as measured by the concentrations of the oxysterols 3β,5⍺-dihydroxycholest-7-en-6-one (DHCEO, Fig. 4B) and 7-DHC 5α,6α-epoxide (ep7DHC, Fig. 4C) that were undetectable in both the sham controls and the HIBI vehicle group.

**Figure 5:**
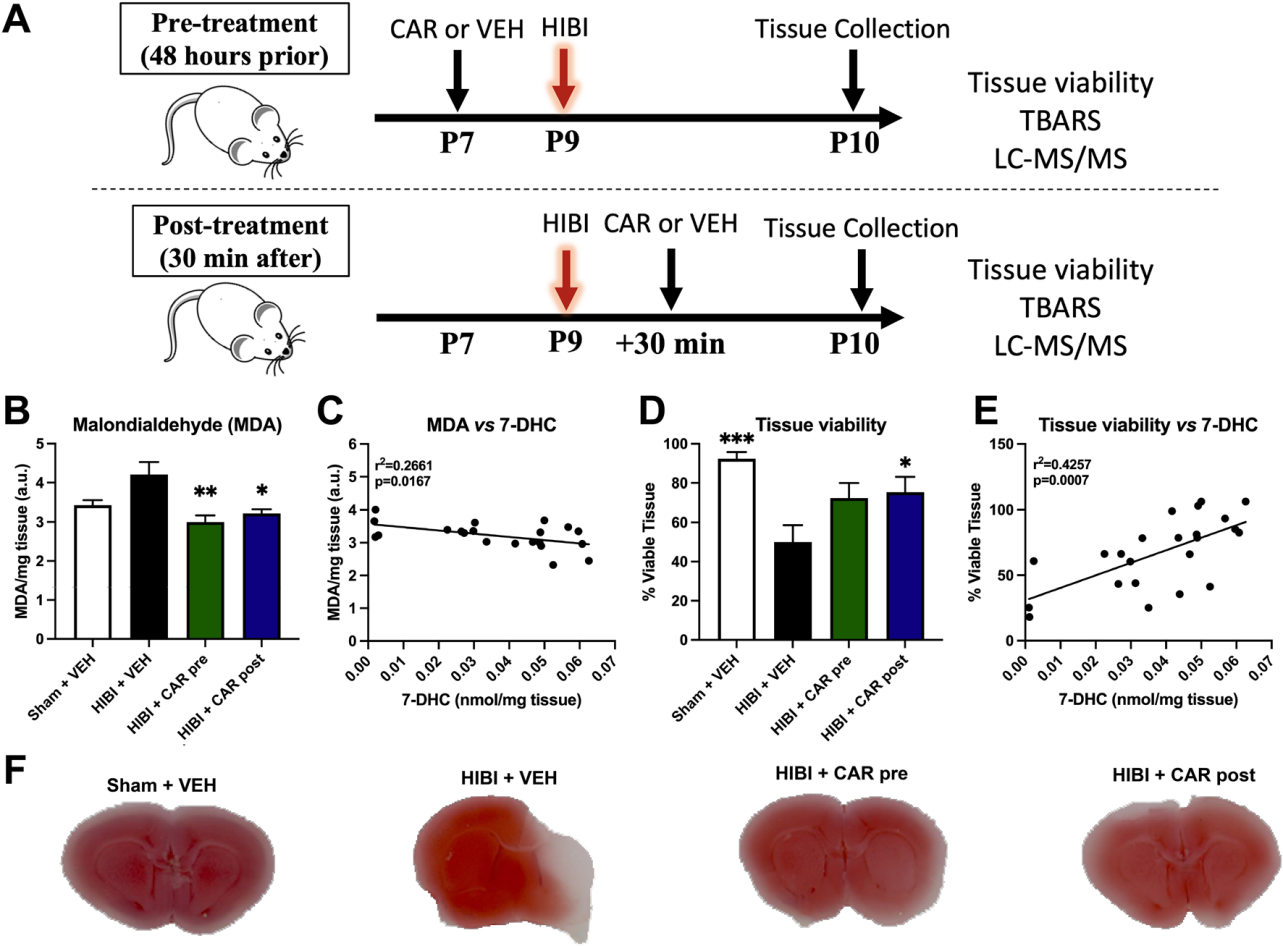
Cariprazine (CAR) pre- or post-treatment both resulted in increased tissue viability and decreased phospholipid oxidation in vivo. (**A**) shows the experimental design. For the pre-treatment experiments, postnatal day 7 (P7) mice were injected with vehicle (VEH) or 0.2 mg/kg CAR 48 h before HIBI. For the post-treatment experiments, mice were injected with vehicle or 0.2 mg/kg CAR 30 min after HIBI; (**B**) MDA levels assessed by TBARS are decreased in animals treated with CAR, indicating that CAR treatment decreases markers of phospholipid oxidation. (**D**) Tissue viability assessed by 2,3,5-Triphenyltetrazolium Chloride (TTC) reveals increased tissue viability in HIBI animals treated with CAR. (**C**) and (**E**) show correlations of MDA vs 7-DHC and tissue viability vs 7-DHC, respectively. (**F**) Representative TTC images for each group. *p < 0.05, **p < 0.01, ***p < 0.001 in pairwise comparison versus HIBI + VEH. Bars represent mean ± SEM of 7–10 replicates.

Additionally, MDA was elevated in the HIBI vehicle group compared to the other three groups (p = 0.004). Both pre- (3.0 ± 0.5 μM MDA/mg tissue, p = 0.002) and post-treatment (3.2 ± 0.3 μM MDA/mg tissue, p = 0.013) with CAR resulted in decreased concentrations compared to HIBI with vehicle (4.2 ± 1.0 μM MDA/mg tissue, Fig. 5B). MDA concentrations were negatively correlated with 7-DHC concentrations in the tissue (p = 0.017, Fig. 5D). Similarly, the percent of viable brain tissue area as measured by TTC staining was significantly decreased in the HIBI vehicle group (50.0 ± 25.9%) compared to sham controls (92.5 ± 10.8%, p < 0.001). Both pre-treatment (72.5 ± 22.4%, p = 0.076) and post-treatment (75.5 ± 22.9%, p = 0.039) with CAR resulted in increased percent of viable tissue area compared to the HIBI vehicle group (Fig. 5C and F), though only post-treatment reached statistical significance with a two-tailed test. Of note, the percent of viable tissue did not differ by sex of the animal for any of the treatment groups (p = 0.454, 0.815, 0.124, and 0.974 for sham vehicle, HIBI vehicle, HIBI pre-treatment with CAR, and HIBI post-treatment with CAR, respectively). The percent of viable tissue was positively correlated with tissue 7-DHC concentrations (p = 0.001, Fig. 5E). Although there was a trend toward higher tissue viability in females with CAR pre-treatment compared to males (80.7 ± 18.6% and 56 ± 23.0%, respectively), this was not significant (p = 0.124), and there was no difference between females (75.9 ± 30.2%) and males (75.3 ± 21.8%, p = 0.974) in the CAR post-treatment group.

## Discussion

Overall, these studies suggest that inhibition of DHCR7 and the resulting increase in 7-DHC induced by CAR improve cell survival in vitro and modulate phospholipid peroxidation in vivo*,* resulting in decreased brain injury after neonatal HIBI. We propose that this protection results from the consumption of the 7-DHC after hypoxia–ischemia, which was supported by the presence of the 7-DHC-derived oxysterols DHCEO and ep7DHC in the mouse brain tissue after CAR treatment but not in the sham or vehicle control groups.

Canonically, ferroptosis occurs due to overwhelming phospholipid peroxidation, which compromises membrane integrity and leads to cell death. The increased amounts of 7-DHC in the membrane (a lipid 10–20 times more oxidizable than polyunsaturated phospholipids)^18,19^ changes the course of lipid peroxidation. Peroxyl radicals produced during the course of lipid oxidation react preferentially with 7-DHC, translating into lower levels of phospholipid peroxidation, ultimately protecting membrane integrity and preventing ferroptosis. We chose to perform the initial RSL-3 ferroptosis studies in the epithelial cell line HT1080 derived from a patient with fibrosarcoma. Although not a brain-derived cell and therefore less directly relevant to neonatal HIBI, many of the original and current studies in ferroptosis^21,26^ were performed in HT1080 cells, and thus we sought to initially test our hypothesis in cells that have consistently shown to be vulnerable to ferroptotic cell death. Our finding that pharmaceutical DHCR7 inhibition protected against RSL-3-induced ferroptosis is consistent with previous reports^21^. Additionally, we demonstrated that the protective effects were associated with an increase in 7-DHC and appeared to be independent of cholesterol levels, as medications such as lovastatin which alter cholesterol concentrations earlier in the biosynthetic pathway did not demonstrate ferroptosis inhibition.

Since hypoxia–ischemia is a complex injury consisting of several types of cell injury and death, it was important to confirm that the protection conferred by CAR-induced 7-DHC elevation was also seen in the OGD model. The use of primary neurons and a pre-established OGD model allowed us to assess if either pre-treatment or post-treatment with cariprazine would affect cell viability, with the hypothesis that pre-treatment with CAR “prepares” the cells/tissues to deal better with the ischemic insults because 7-DHC is already elevated when the injury occurs, and that this elevation changes the course of lipid peroxidation and associated ferroptosis cell death. The positive results from the neuron OGD treatment were consistent with several previous studies that have demonstrated a significant role of ferroptosis in neuronal OGD^27–29^.

To investigate the neuroprotective benefits of CAR in vivo, we used a pre-established mouse model for HIBI. In mice treated with CAR there was a significant increase in 7-DHC in the brain, confirming previous observations from our group^30^. It is known that neonatal HIBI leads to increase in phospholipid peroxidation (as demonstrated by elevated MDA concentrations) and a decrease in tissue viability^31–34^, an observation consistent with ferroptotic cell death. Treatment with CAR in our study decreased MDA and increased tissue viability, indicating that both pre- and post-treatment with CAR suppresses lipid peroxidation – the main mechanism of ferroptotic cell death. At the stage of brain maturity used in this model (postnatal day 9, which correlates to the brain maturity of a term human newborn), sterol biosynthesis is highly active in the brain, which allows for a rapid accumulation of 7-DHC when the enzyme is inhibited^35,36^. Although it could be reasoned that a more significant benefit would be seen with pre-treatment so that 7-DHC concentrations are already elevated at the time of injury, pre-treatment is unfortunately not clinically relevant since clinicians are currently unable to predict prior to delivery which infants will have HIBI. Additionally, our data indicate that post-treatment with CAR 30 min after HIBI was at least as effective as pre-treatment, suggesting that this potential intervention can be administered at clinically relevant time points.

Post-treatment with CAR resulted in significantly lower MDA concentrations and larger areas of brain tissue viability despite a non-significant increase in 7-DHC. This could suggest that even a small increase in 7-DHC levels during the onset of lipid peroxidation is enough to protect phospholipids and tissue integrity. An additional explanation for the non-significant increase in 7-DHC is that 7-DHC produced after HIBI is rapidly oxidized, not allowing for the accumulation seen with pre-treatment. This latter hypothesis is supported by the presence of the 7-DHC derived oxysterols DHCEO and ep7DHC in the CAR treated groups when they could not be detected in the sham or HIBI vehicle brains. Although it is a limitation of the current study and further investigation into the time-specific changes in 7-DHC and oxysterols after injury will be important, the current results suggest fairly rapid oxidation (within the first 24 h). This is important, as it is well known that a chronic accumulation of 7-DHC is deleterious and should be avoided^36,37^. Although there are other potential mechanisms that cannot be excluded by the current study, we postulate here that during conditions like HIBI where oxidative stress is increased and overwhelming lipid peroxidation damages tissues, briefly increasing 7-DHC only during the acute period of oxidative injury may be protective.

The current studies were designed using one of the most potent DHCR7 chemical inhibitors, cariprazine. We used it as a proof of principle to highlight that 7-DHC-elevating medications can be a potential therapeutic approach for ischemic-reperfusion injuries such as HIBI. Of note for neonatal medicine, several medications that are commonly used in neonates were also found to significantly increase 7-DHC concentrations in vitro; these include vitamin D – or its active form, calcitriol (CAL) – and levetiracetam^24^. Additionally, vitamin D was recently shown to inhibit ferroptosis in the rat model of HIBI^38^. Although these medications are not as potent as CAR, they may be used either individually or as combined treatments to acutely increase 7-DHC in neonatal HIBI, which should be further investigated.

In conclusion, our studies show that elevating 7-DHC concentrations with FDA-approved psychotropic medications with DHCR7-inhibiting effects can improve cellular and tissue viability after HIBI. Further studies are needed to confirm the neuroprotective findings and clarify the exact mechanisms of neuroprotection, including assessing whether some of the primary effects of CAR, such as alteration in serotonin and dopamine, may play a role. Overall, our results strongly suggest that 7-DHC elevation in the brain may be an effective novel therapeutic mechanism in neonatal HIBI.

## Materials and methods

### Chemicals

Unless otherwise noted, all chemicals were purchased from Sigma-Aldrich Co (St. Louis, MO). HPLC grade solvents were purchased from VWR BDH chemicals (Radnor, PA). All sterol standards, natural and isotopically labelled, used in this study are available from Kerafast, Inc. (Boston, MA). RSL-3 was purchased from Sigma-Aldrich (St. Louis, MO).

### HT1080 cell line cultures

Since many of the original studies in ferroptosis^26^ were performed in HT1080 cells that consistently demonstrate vulnerability to ferroptotic cell death, we initially tested our hypothesis in the HT1080 cell line. Human fibrosarcoma (HT1080) cells were acquired from ATCC.

### RSL3-induced ferroptosis injury

RSL-3 ((1S,3R)-RSL-3) is a potent ferroptosis activator. RSL-3 binds and inactivates GPX4 and mediates GPX4-regulated ferroptosis. After plating cells in regular medium, RSL-3 was mixed with fresh medium and 25 nM RSL-3 was added to cell cultures 24 h prior to cell death analyses (Fig. 2A).

### Primary neuronal cultures

Primary cortical neuronal cultures were prepared from embryonic day 18 mice as previously described^35^. Briefly, the brain was placed in pre-chilled HBSS solution (without Ca^2+^ or Mg^2+^), and two cortices were dissected and cut with scissors into small chunks of similar sizes and transferred to Trypsin/EDTA (0.25%) for 25 min at 37 °C. Trypsin was inactivated by adding Trypsin Inhibitor (Sigma T6522) for 5 min. Samples were then spun at 100 g, the tissue resuspended in Neurobasal medium with B-27 supplement (Gibco #17504-044) and then triturated with a fire-polished Pasteur pipette. The cells were pelleted by centrifugation for 5 min at 80 g. The cell pellet was resuspended in Neurobasal medium with B-27 supplement and the cells counted. The cells were plated on poly-d-lysine coated 96-well plates at 60,000 cells/well. The growth medium was Neurobasal medium with B-27 supplement without antioxidants and Glutamax. Cells were incubated at 37 °C, 5% CO_2_.

### Drug treatment in cell cultures

The drug treatment timing and dose determination for the in vitro studies were based on our previous studies in Neuro2a and primary cortical neuronal cultures^24,25,39,40^. Our previous studies demonstrated that the drug concentrations resulting in maximum 7-DHC increase after 24 h without negatively affecting cell viability were 25–50 nM CAR, 500 nM ARI, 500 nM TRZ, and 50 nM lovastatin (LOV), so these were used as the concentrations for the initial HT1080 RSL-3 study, and as the maximum dose for the dose dependent CAR studies.

### OGD injury

Primary cortical neurons underwent OGD injury on day in vitro 6. The OGD model simulates hypoxic-ischemic injury by replacing the media with glucose- and pyruvate-deficient media and placing cells in a tri-gas incubator at 37 °C, 1% oxygen, 5% CO_2_, and 94% nitrogen. After 4 h of OGD, cells were placed back in the normoxic incubator at 37 °C and 5% CO_2_, and glucose and pyruvate re-introduced to the media for the reperfusion period of 24 h (Fig. 3A). Control cells remained in normoxic culture with glucose-rich media at 37 °C and 5% CO_2_ for the same duration.

### Cell viability assay

Cell viability was assessed 48 h (unless stated otherwise) after injury using alamarBlue as an indicator of viable cells. alamarBlue solution was made by dissolving of 1 g resazurin sodium salt in 100 mL sterile PBS and sterile filtrated through a 0.22 µm filter. Stock solutions were stored at 4 °C. The working solution was made by adding 200 µL of the stock solution to 50 mL culture medium. After 2–4 h of incubation, viability was estimated by measuring the fluorescence using a 540/35 excitation filter and a 590/20 emission on a SpectraMax Plus 384 microplate reader (Molecular Devices).

### Mouse model of neonatal HIBI

This study was approved by the University of Nebraska Medical Center Institutional Animal Care and Use Committee, and all methods were performed in accordance with the relevant guidelines and regulations. The in vivo studies are reported in accordance with ARRIVE (Animal Research: Reporting of In Vivo Experiments) guidelines. Postnatal day 9 CD1 pups were anesthetized using 2.5% isoflurane and were randomized by random number generation to either unilateral carotid artery ligation or sham surgery (carotid dissection without ligation). The investigators performing the tissue analyses were blinded to the group allocation. The CD1 strain was chosen due to a high rate and level of brain injury with low mortality rate compared to other mouse strains^41^. Due to the known therapeutic effect of hypothermia on neonatal HIBI, all procedures were performed at normothermia using rectal temperature probes and thermal pads. After two hours of recovery, the injury group underwent 30 min of hypoxia at 8% oxygen in a hypoxia chamber (Biospherix). The sham group was also separated from the dam for 30 min but remained at 21% oxygen.

Animals were randomized to one of four treatment groups (n = 9/group): (1) sham surgery + normoxia + vehicle (sham control); (2) HIBI + saline (vehicle control); (3) HIBI + 0.2 mg/kg cariprazine 48 h prior to injury (pre-treatment); or (4) HIBI + 0.2 mg/kg cariprazine 30 min after injury (post-treatment). The dose of 0.2 mg/kg in mouse corresponds to the lowest dose used in humans (1.5 mg tablet/day). Animal equivalent dose was calculated based on previously published calculations for conversion^42^ and validated in our previous study^30^. Animals were sacrificed and brain tissue extracted 24 h after injury (Fig. 5A). For biochemical analysis, ipsilateral brain tissue was rapidly dissected and flash frozen prior to homogenization for analysis.

### Brain infarct and phospholipid peroxidation assays

As a general measure of overall injury severity, infarct area was assessed by staining coronal sections with 2,3,5-Triphenyltetrazolium chloride (TTC). The brain was removed and 1 mm coronal sections were obtained. The sections were placed in 2% TTC solution and incubated at 37 °C for 20 min, followed by fixation in formalin overnight. Stained sections were scanned and digitized, and Image J software (NIH) was used to estimate the percent of ipsilateral viable tissue (ipsilateral stained area/contralateral total area).

For biochemical measures, immediately after removing the brain, the hemispheres were separated and the ipsilateral tissue flash frozen for future processing. Prior to malondialdehyde (MDA) or sterol analyses, the frozen tissue was sonicated in ice cold phosphate buffered saline. Measurement of the phospholipid peroxidation marker MDA was conducted using the thiobarbituric acid reactive substances (TBARS) assay as described previously^43^.

### Lipid extraction and sample preparation

In cell culture experiments, for cells plated in 96-well plates, sterol levels were analyzed in individual wells and final reported values represent the mean of 8–12 technical replicates. After rinsing plates with 1X PBS, 200 μL of methanol containing the internal standard was added, as reported previously^44^. The plate was placed on an orbital shaker for 30 min at room temperature and centrifuged for 10 min using a Sorvall swing rotor. An aliquot (100 µL) of the supernatant was transferred to a 4-Phenyl-1,2,4-triazoline-3,5-dione (PTAD)-predeposited plate, sealed with Easy Pierce Heat Sealing Foil followed by 30 min agitation at room temperature, and analyzed by liquid chromatography with tandem mass spectrometry (LC–MS/MS).

### LC–MS/MS measurements of cholesterol, 7-DHC and oxysterols

For cell culture sterol analyses, at 24 h after RSL-3 or OGD injury, 10 *μ*L of Hoechst dye was added to all wells in the 96-well plate, and the total number of cells counted using the ImageXpress Pico Automated Cell Imaging System (Molecular Devices, San Jose, CA). After removing the media, wells were rinsed twice with phosphate-buffered saline, 10 µl of BHT/TPP antioxidant was added to each well and the plates were stored at − 80 °C for lipid analysis.

Extracted sterols were derivatized from cells or tissue homogenate with PTAD as described previously^25,45^ and placed in an Acquity UPLC system equipped with ANSI-compliant well plate holder coupled to a Thermo Scientific TSQ Quantis mass spectrometer equipped with an APCI source. Then 5 μL was injected onto the column (Acquity BEH C18 1.7 μm, 2.1 mm × 50 mm) with 100% methanol (0.1% v/v acetic acid) mobile phase for 2.0 min runtime at a flow rate of 500 μL/min. Natural sterols were analyzed by selective reaction monitoring using the following transitions: Chol 369 → 369, 7-DHC 560 → 365, desmosterol 592 → 560, lanosterol 634 → 602, with retention times of 0.7, 0.4, 0.3 and 0.3 min, respectively. 7-DHC-derived oxysterols were analyzed as described previously^17,30^.

### Statistical design

To minimize Type I error, group comparisons were performed by one way ANOVA for analyses involving multiple doses of drug or multiple time points for drug administration. For statistically significant groupwise comparisons, pairwise comparisons were performed using Dunnett’s test with p values adjusted for multiple comparisons. Correlations between 7-DHC concentrations and MDA or tissue viability were performed by linear regression analyses. For the hypothesis-generating in vivo experiments, sample size was determined by the Three Rs principle to minimize the number of animals while taking into account the variability in injury severity in the mouse HIBI model. All tests were two-tailed. Statistical analyses were performed using GraphPad Prism version 9.5.1.

## Data Availability

The datasets generated and analyzed during the current study are available in the Figshare repository, 10.6084/m9.figshare.23535510.
